# Interactional practices accomplished by index-finger pointing directed at the addressee in Hebrew face-to-face interaction

**DOI:** 10.3389/fpsyg.2024.1463449

**Published:** 2025-01-15

**Authors:** Anna Inbar

**Affiliations:** Department of Hebrew Language, Levinsky-Wingate Academic College, Tel Aviv, Israel; University of Haifa, Haifa, Israel

**Keywords:** pointing, attention-drawing device, multimodal stance-taking, negative stance, epistemic authority

## Abstract

This study uses Hebrew data to examine the practices accomplished by index-finger pointing toward the addressee, with a focus on interactional purposes beyond merely indexing the reference. The data were taken from the *Haifa Multimodal Corpus of Spoken Hebrew*, which consists of video recordings of naturally occurring casual conversations collected between 2016 and 2023. By employing the methodologies of interactional linguistics and multimodal conversation analysis, the study elaborates on the social actions that are accomplished via this gesture, showing that pointing at the addressee in Hebrew talk-in-interaction can be explained from different perspectives. The study suggests that non-referential pointing primarily serves as an attention-drawing device. However, similar to other gestural or verbal attention-drawing devices, in some contexts, the gesture can also be considered to be a cue whereby conveying a negative stance or displaying epistemic authority is recognized. Additionally, it can be employed as an abrupt way of interrupting or as an attempt to elicit a response from the addressee.

## 1 Introduction

The prototypical pointing gesture (*cf. pure pointing*, Kendon, 1980; *pointing-out*, Lakoff, 1987; *canonical pointing*, Langacker, 2008) is a bodily movement toward a target in order to direct someone’s attention to it (e.g., Eco, 1976, p. 119; Clark, 2003; Cooperrider, 2023; Cooperrider et al., 2018; Kendon, 2004; Kita, 2003). The target of pointing is presumed to be visual and present in the speech situation; if the pointing gesture is co-produced with speech, it is taken for granted that what is pointed to is in some sense identical to what is simultaneously referred to in speech (*cf.* Clark et al., 1983). An extended index finger is often considered to be the origin of pointing and is connected to the target via an imaginary line or trajectory (e.g., Enfield, 2009; Kita, 2003; McNeill et al., 1993). However, the preference for pointing with the index finger is not universal (e.g., Cooperrider et al., 2018; Wilkins, 2003), and pointing comprises a much broader range of bodily actions involving the thumb, hands, extended arm, head, face (e.g., lip-pointing), and objects (e.g., Cooperrider, 2023; Kendon, 2004). Moreover, even within cultures in which the gesture prototypically takes the form of an extended index finger, other forms can also be used, potentially revealing functional differences (*cf.* Kendon, 2004). The gesture is usually characterized by a “post-stroke hold” (Kita et al., 1998) or “stasis” (Cooperrider, Forthcoming), a brief visual suspension of the gesture from a dynamic to a static position before being retracted or beginning the next gesture (*cf.* Kendon, 1980, 2004; Bressem and Ladewig, 2011).

Whereas prototypical pointing is used to make a reference to entities that are physically present in the immediate space of the interaction, such as an object, a person, a location, or a direction (e.g., Clark, 2003; Kendon, 2004; Kita, 2003), pointing can also be directed toward a seemingly empty space to provide new references (e.g., *abstract deixis*, McNeill, 1992; McNeill et al., 1993; *Deixis am Phantasma*, Stukenbrock, 2014). In addition, the pointed-at object can metonymically represent the intended (discourse) referent (e.g., Haviland, 2000; Levinson, 2006). As noted by Cooperrider (2014), such metonymic pointing is relatively well attested in the ethnographic literature on pointing, leading researchers to suggest that this type of metonymy “may be a pervasive feature of pointing in real-world settings” (p. 3).

While some studies of pointing gestures have addressed their deictic referential function (e.g., Clark, 2003; Kendon, 2004; Kita, 2003), other studies have revealed interactional practices that are accomplished by pointing (e.g., Bangerter, 2004; Cooperrider, 2011, 2014, 2016; Enfield et al., 2007; Goodwin, 2003; Healy, 2012; Hindmarsh and Heath, 2000; Holler, 2010; Mondada, 2007, 2012, 2014; Streeck, 2017; Yasui, 2023). For example, Mondada (2007) observed a work meeting involving a team of agronomists and computer scientists, and noted that the speakers used pointing gestures directed toward maps and other documents as a turn-taking device: In the turn-initial position, pointing indicated incipient speakership; in the pre-turn-initial position, pointing could be used as a claim for the next turn before the prior turn had been completed.

The trajectory of a pointing gesture can single out one of the participants in a conversation. As prototypical pointing is presumed to bring the recipient’s attention to a pointed-at entity (e.g., Kita, 2003), the pointing at the addressee raises some questions, such as under what circumstances the speaker would request the addressee to pay attention to themselves, or whether the addressee is the true target of the pointing. Several studies have shown that a pointed-at co-participant can stand in an (apparent) metonymic relation of speaker for utterance (e.g., Ishino, 2009); for example, when the speaker cites what the conversational participant has just said (e.g., Bavelas et al., 1992). Other studies have shown that English-speakers may use a pointing gesture to indicate agreement with a pointed-at person (e.g., Healy, 2012). Some scholars (e.g., Bavelas et al., 1995; Enfield et al., 2007) have suggested that, in the course of interaction, the gesture can be used to specify the addressee of an utterance in order to elicit their response. However, pointing at the addressee appears to be a frequent phenomenon in dyadic face-to-face interactions (as the current study attests), in which singling out a co-participant as the intended recipient of a certain action is irrelevant.

The current study explores interactional practices, beyond indexing the reference, that are accomplished by index-finger pointing directed at the addressee by Hebrew speakers. The following sections first introduce the data and methodology (section 2). Subsequently, in order for the target phenomenon to emerge as a distinct one, several cases in which pointing at the addressee is used for deictic referential functions are provided (section 3). Non-referential pointing at the addressee—the cases in which the relationship between what is pointed at and what is said is not straightforward—is then elaborated on (section 4). Following this, the findings are discussed, showing that these pointing gestures appear to differ functionally from the canonical case (section 5). Finally, the study is summarized, and concluding remarks are provided (section 6).

## 2 Data and methodology

The data are drawn from the *Haifa Multimodal Corpus of Spoken Hebrew* (Maschler et al., 2024), which comprises approximately 22 h of video recordings of naturalistic conversations among friends and family members that were collected between 2016 and 2023. Informed consent for the collection and publication of the data was obtained from all of the participants. The participants were filmed in natural settings, including their homes, cafés, and workplaces, during casual conversations of approximately 30 min to 2 h. Following the setup of the camera and recording device, the researcher exited the environment, allowing the participants to interact freely without providing any instructions. This design facilitated uninstructed dialogue, enabling the participants to discuss the topics of their choice and fostering authentic social interaction. The present study is based on approximately nine and a half hours (571 min) of talk from 13 conversations—involving 30 speakers in total (nine dyadic, two triadic, and two quadratic conversations)—that were recorded between 2017 and 2021.

Tokens of addressee-directed index-finger pointing gestures were searched manually, excluding those used for indexing reference, such as when they were coordinated with utterances that included indexing the second person or when the pointed-at co-participant stood in an (apparent) metonymic relation of speaker for utterance or performer for action (see Section 3). The collection of addressee-directed index-finger pointing gestures, produced without any verbal reference to the addressee and not analyzed as metonymic pointing, comprised 81 tokens. These findings reveal that non-referential pointing directed at the addressee occurred at least every 5.3 min on average; ambiguous cases, such as those involving indexing the second person while also occurring in the contexts found to be associated with non-referential pointing, were not included in this count.^1^

To elaborate on the actions that were accomplished via such gestures, I employ the methodologies of interactional linguistics (Couper-Kuhlen and Selting, 2018) and multimodal conversation analysis (e.g., Goodwin, 2018; Mondada, 2016). The analysis will consider the position of the gesture within turn and sequence, the accompanying talk, other bodily conduct, the surrounding environment, and the semiotic properties of the gesture—all of which may combine to contextualize the practices accomplished by the gestures at issue.

From the morphological perspective, the addressee-directed pointing gestures were identified by “movement toward” (Eco, 1976, p. 119) the addressee, using an extended finger. Usually, the index-finger morphology was used; however, in rarer cases, pointing at the addressee was accomplished using other fingers, such as the ring finger, while the little finger was also extended (one token), and the little finger (two tokens). These ring-finger and little-finger pointing gestures can be considered as *ad hoc* forms of pointing that in both cases were driven by “biomechanical ease” (*cf.* Cooperrider, 2024). The ring-finger pointing appeared when the speaker held a glass in her hand; therefore, it seems that, from the physiological perspective, the index finger (together with the middle finger) was preferred for holding the glass. The little-finger pointing occurred in an interaction involving four participants, and seemed to be influenced by the participants’ seating arrangement. This gesture was produced using the little finger of the right hand when the speaker turned to the participant sitting next to him from the right side. It seems that, in this condition, performing the gesture using the little finger took less effort since the speaker only needed to move the finger slightly to the right; had he pointed using his index finger, he would have had to move his entire hand.

When the index-finger morphology was used, the index finger was clearly protruded more than any other finger (in some cases the middle finger was also extended), with the thumb and other fingers remaining flexed. Flexing and extending varied in degree. Usually, index-finger pointing occurred with pronated orientation, but six times it occurred with supinated orientation. The gesture was produced by pivoting the arm from either the shoulder or from the elbow. Although such morphological differences may have an impact on the interaction between participants (*cf.* Holler, 2010; Kendon, 2004) and reveal further form-function correlations, the wide morphological variety found in the data revealed only few examples of each type, thus making meaningful quantitative comparisons difficult to accomplish.

## 3 Referential pointing at the addressee

Referential pointing occurs when the reference to the pointed-at participant is part of the propositional content. In such cases, the gesture is often coordinated with an utterance that includes indexing the second person. Such pointing is illustrated in Excerpt 1, which is taken from a conversation between two friends, Sigal and Orly. Prior to the segment shown in Excerpt 1, Orly told Sigal that she would like to go the United States, but her visa had expired.

**EXCERPT 1:**
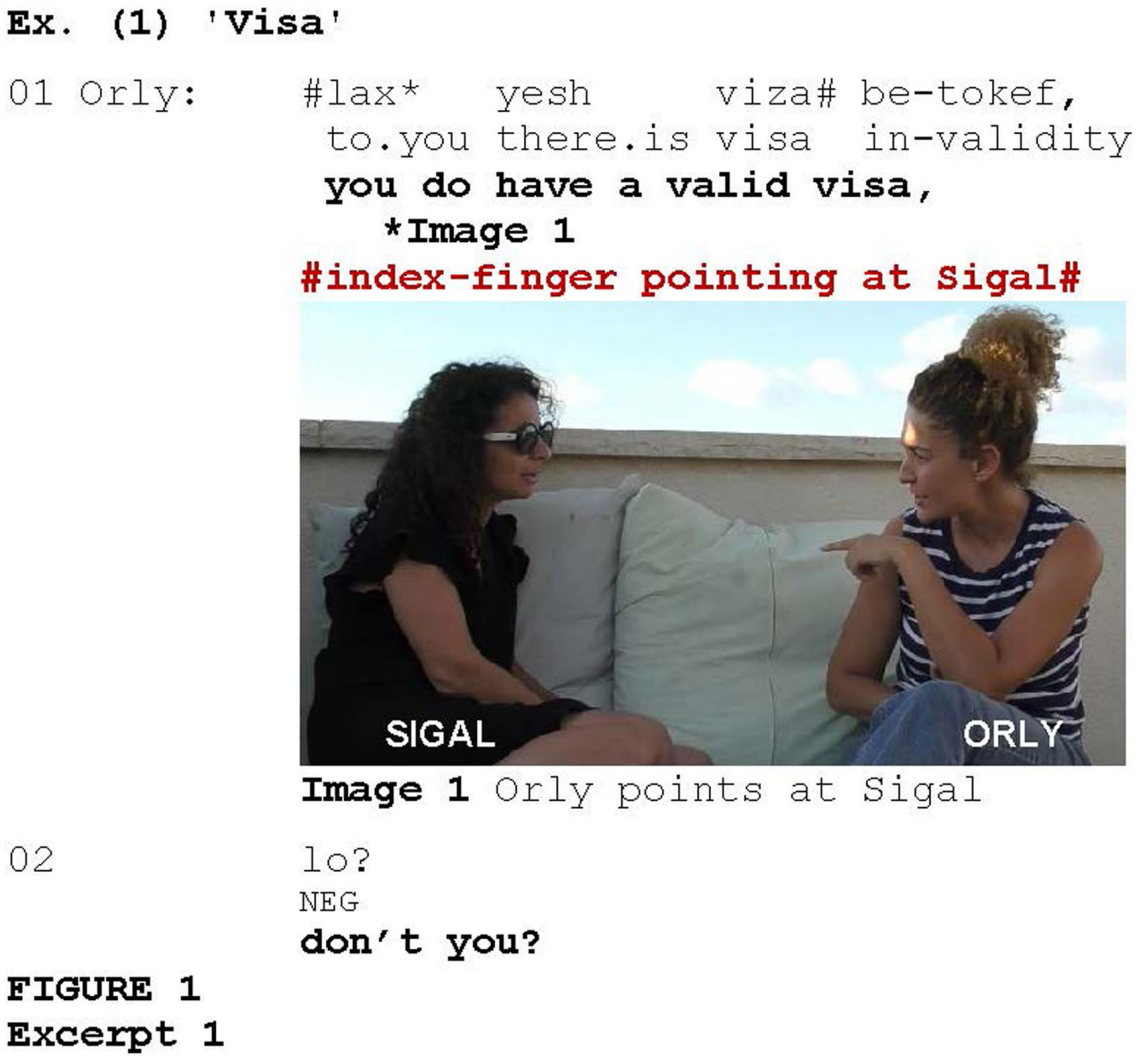


Coordinated with the index-finger pointing at Sigal (Image 1), Orly asks Sigal whether she has a valid visa (lines 1–2). Orly’s utterance includes a verbal reference to Sigal in the form of the second person dative pronoun *lax* “to you” (line 1); thus, such pointing can be considered to be referential. However, this is an ambiguous case, since the pointing occurs in the context of eliciting a response, in which the gesture was also found to be used without any verbal reference to the second person, as will be shown in Section 4.

Referential pointing can also occur in cases in which the addressee stands in a metonymic relation of speaker for utterance or speaker for action. As in prototypical pointing a pointed-at object is usually under a particular description (Clark, 2003, p. 247), the content of the addressee’s utterance or the action that they perform is often evaluated by a pointer in a verbal component that is co-produced with the pointing gesture.

Excerpt 2 illustrates metonymic pointing in which the addressee represents the content of her previous talk. The example is taken from a conversation between two friends, Naomi and Kelsey. Prior to the segment in Excerpt 2, Kelsey told a story about a couple, friends of hers, who had a big celebration of their marriage proposal as if the proposal was happening in real time. However, the actual proposal had already been made previously and the couple had even set a date for the wedding. Kelsey and Naomi try to understand why they needed to have such an event. Kelsey says that maybe it had something to do with the ring (lines 1, 3); Naomi points at Kelsey with her index finger (Image 2) as she utters *ze mamash muzar* “that is really weird” (line 4), evaluating the story that Kelsey told as being extremely odd. In this case, the *discourse deixis* (Cornish, 2011, 2012) is accomplished not only gesturally, but also verbally by deploying the relative pronoun *ze* “this.”

**Figure:**
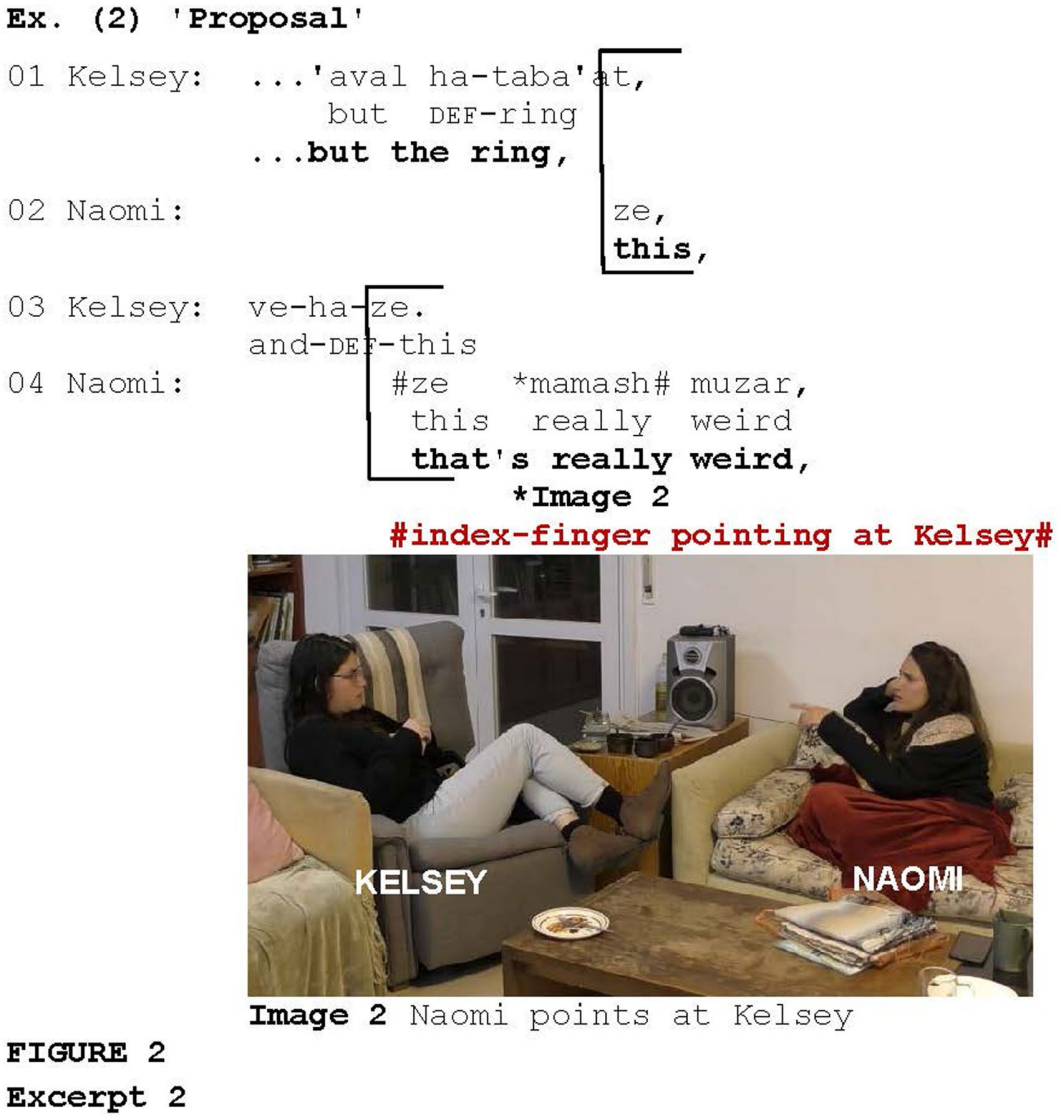


In Excerpt 3, the index-finger pointing occurred when the pointer treated the addressee’s way of behaving with ridicule. The example is taken from a conversation involving a couple, Alon and Hillel. Alon is sitting with their baby cradled in his arms. After 37 min of conversation, their friend, Einav, joins them. We enter the interaction after Einav has interacted with Hillel and Alon for about six and a half minutes.

**Figure:**
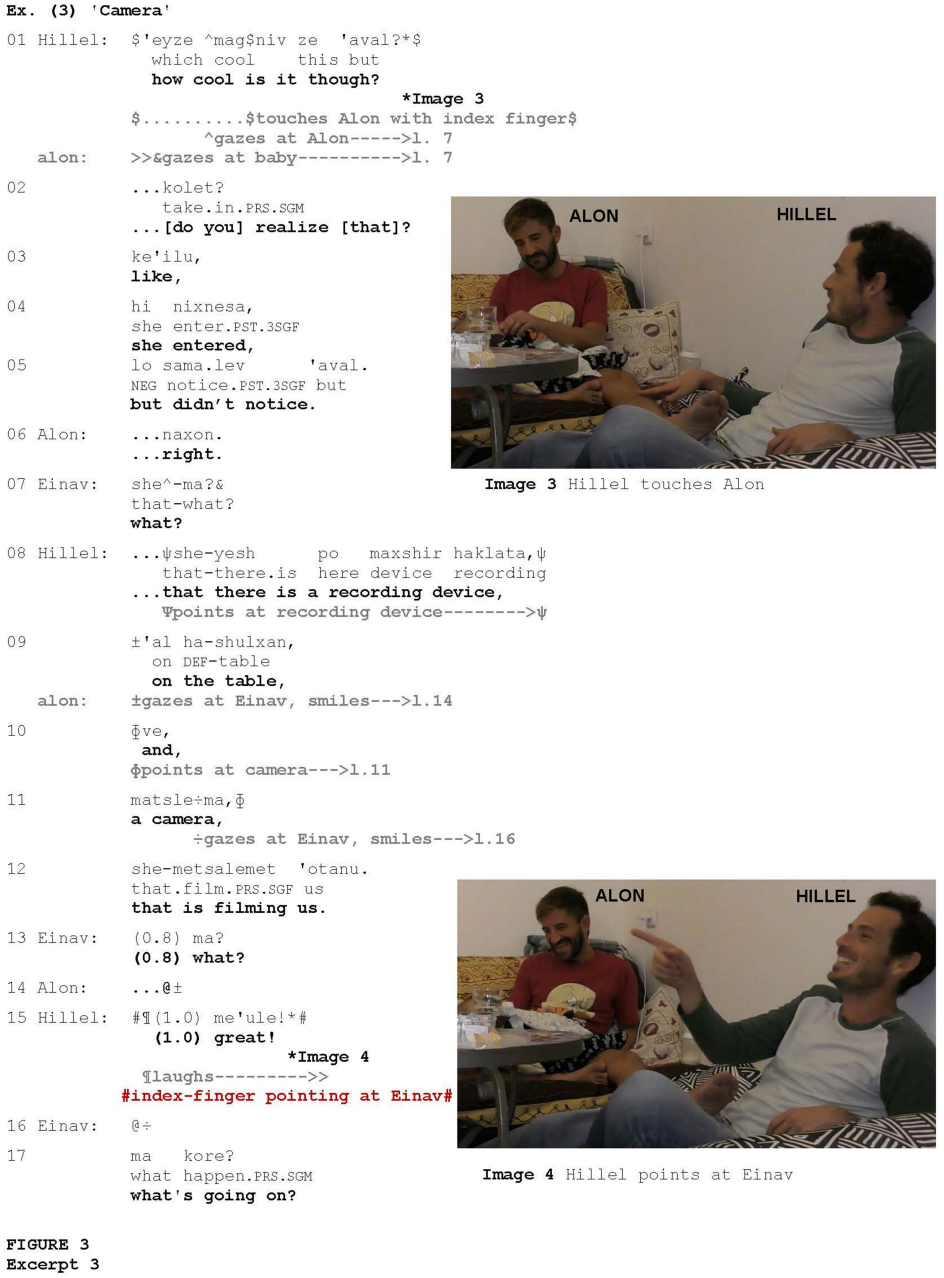


Hillel addresses Alon via a question, also attracting his attention via touch (Image 3), expressing surprise that Einav did not notice [that a camera and a recording device were in the room] (lines 1–5). Alon confirms this via *naxon* “right” (line 6) and Einav asks what it is that she did not notice (line 7). Hillel explains that a camera and a recording device are in the room, and points to these objects (lines 8, 10–11). Einav conveys surprise via *ma?* “what?” (line 13), while Hillel and Alon gaze at her and smile. Hillel and Alon then start laughing, and Hillel points at Einav with his index finger (Image 4). By pointing at Einav, Hillel metonymically spotlights her behavior, locating it as the cause of laughter (*cf.* Yasui, 2023) and evaluating it verbally as great (line 15).

In Excerpts 2 and 3, the pointed-at participants metonymically represented what they had said previously or were currently doing, respectively. Such examples of target-referent metonymy can be characterized as involving a “chain of indicating” (Clark, 2003, p. 264), namely static structures that can be examined link by link, in which the speaker indicates the addressee which, in turn, indexes the referent—utterance or action. However, Cooperrider (2014) argues that, from a cognitive perspective, the target-referent metonymy may be better characterized as being driven by *compression* (Fauconnier and Turner, 2002), which is a feature of conceptual integration. In this case, the pointed-at participants and their actions or utterances are connected via a conceptual relation.

The detailed description and distribution of various types of referential pointing directed at the addressee will be addressed in future research. This paper will now shift its focus to non-referential pointing, which constitutes the central concern of the current study.

## 4 Non-referential pointing at the addressee

The contexts in which the non-referential index-finger pointing directed at the addressee were employed appeared to be diverse. In the majority of the cases (*N* = 41), the gesture occurred in contexts that shared a broad sense of opposition: some were disaffiliative contexts associated with dispreferred actions, such as disagreement, disconfirmation, and repair, while in others speakers conveyed information that was (assumed to be) contrary to the addresses’ expectations. In these contexts, speakers typically established and maintained a convergent status and stance of *epistemic authority* (Heritage, 2012). In other contexts, recipients displayed cues of disengagement in the interaction (*N* = 15) or speakers attempted to elicit a (minimal) response from the addressee (*N* = 13). Additionally, some occurrences followed the completion of a cognitive process (*N* = 7), typically related to remembering, while others were associated with interruptions and discourse suspension (*N* = 5). Sometimes it was challenging to delineate among these categories, as some occurrences fit into more than one category.^2^ In what follows, I will illustrate these contexts.

### 4.1 Disconfirmation

In Excerpt 4, the gesture is associated with disconfirmation. Prior to the excerpt, Dotan told Alex that he had attended a concert held at the singer’s house. After Dotan explained exactly where the concert took place, Alex asks whether everyone there is a musician (line 1). Dotan disconfirms this via *lo* “no” (line 2), stating that at least one hairdresser lives there (line 3), while pointing at Alex with his index finger (Image 5). He later explains (not shown) that this knowledge was based on the fact that his wife once visited that hairdresser.

**Figure:**
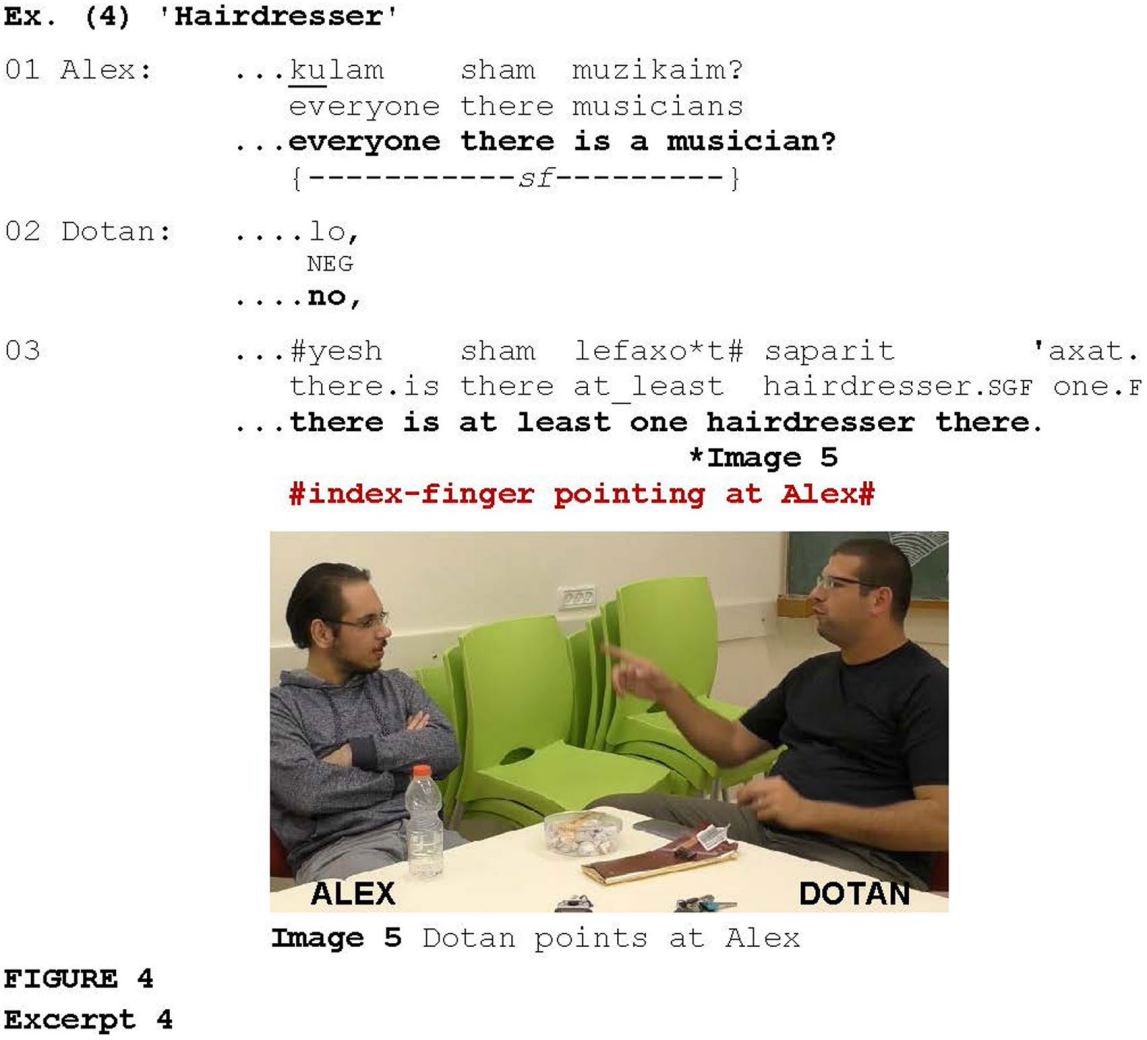


### 4.2 Disagreement

In Excerpt 5, the gesture is associated with disagreement. The excerpt is taken from a conversation between two friends, Dov and Boaz, which revolves around politics and the controversial status of the current Israeli Prime Minister. In Excerpt 5, Boaz disagrees with Dov’s definition of democracy and then explains why.

**EXCERPT 5:**
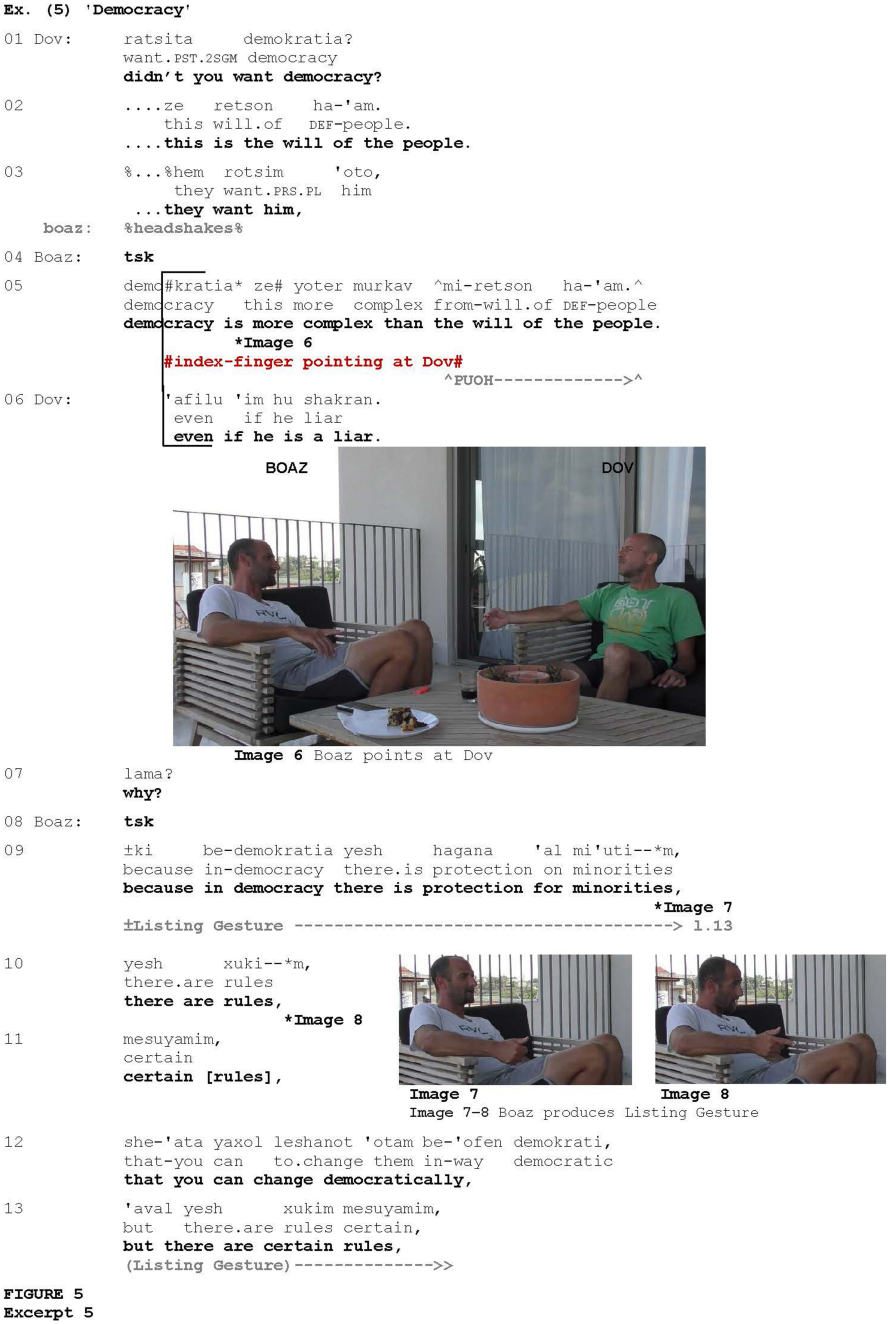


Dov claims that the tenure of the current Israeli prime minister is a consequence of democracy, which is the will of the people (lines 1–3). In response to Dov’s definition of democracy as the will of the people (line 2), overlapping with Dan, Boaz expresses disagreement via headshakes (line 3). Then, prefaced by a click (line 4) expressing a negative stance (Ben-Moshe and Maschler, Forthcoming), Boaz proceeds to express disagreement verbally, saying that democracy is more complex than the will of the people (line 5). This utterance is associated with two pragmatic gestures: Boaz first briefly points at Dov with his index finger (Image 6) and then performs the Palm Up Open Hand gesture which is often used to frame a content as obvious, self-evident, or as shared knowledge (e.g., Inbar and Maschler, 2023). Dov requests explanation (line 7), thus admitting Boaz’s *epistemic authority* (Heritage, 2012) on the subject. In response, Boaz provides a list of explanations (lines 9–13), coordinated with a particular type of listing gesture—the Finger-counting gesture (Images 7 and 8), which tends to appear in contexts of opposition and is associated with epistemic authority among Hebrew speakers (Inbar, 2020; Inbar, Forthcoming).

### 4.3 Displaying epistemic authority

Interestingly, a demonstration of knowledge was observable in most of the cases in which the index-finger pointing directed at the addressee occurred in disaffiliative contexts or when the addressee did not expect the information that was provided. This was the case in Excerpts 4 and 5. In Excerpt 4, Dotan was the person who knew who lived in the place that was being discussed, and Alex addressed him by asking a question regarding this issue. In Excerpt 5, Boaz positioned himself as being more knowledgeable about politics when he disapproved of Dov’s definition of democracy, and Dov addressed Boaz with the question “why?” Moreover, Boaz deployed the Finger-counting gesture, which is another strategy that is found in contexts in which speakers produce and maintain convergent status and stance of epistemic authority (Inbar, 2020, 2024).

More striking examples of a display of epistemic authority are illustrated in Excerpt 6, which is taken from a conversation between two friends, Kelsey and Naomi. The participants discuss the use of disposable utensils. Prior to what is shown in Excerpt 6, Kelsey said that such use was unreasonable, but that she could understand her mother using disposable utensils when having many guests. She adds that in such cases, her mother should use disposable utensils made of paper (lines 1–3). In what follows, Naomi, who is currently attending a textile school, explains that most disposable utensils made of paper cannot decompose or be recycled.

**Figure:**
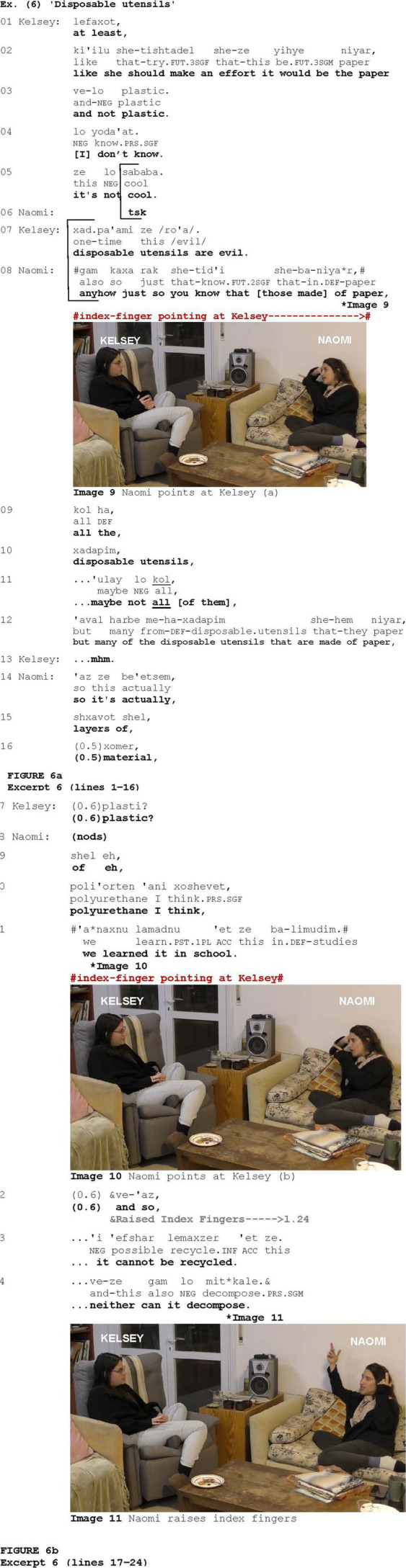


In response to Kelsey’s statement that it is better to use disposable utensils made of paper (lines 1–3), Naomi produces a click (line 6), projecting disaffiliation, and she will later explain that such material cannot actually decompose or be recycled (lines 22–24). Prefaced by two discourse markers—*gam kaxa* (lit.) “also so” conveying concession or returning to an earlier subject after a digression, and *rak she-tid’i* “just so you know/FYI” conveying epistemic authority (line 8), Naomi raises the issue of disposable utensils made of paper for further discussion (lines 8–12). When she utters *gam kaxa rak she-tid’i she-ba-niyar*, “anyhow just so you know that [those made] of paper,” she points at Kelsey with her index-finger (Image 9). Although the verbal form *tid’i* consists of the reference to the second person, the gesture occurs in the opposing context and is associated with epistemic authority by virtue of being co-produced with such a phrase. Naomi then projects a violation of expectation via *be’etsem* “actually” (line 14) and begins to explain about the materials of which such utensils are made (lines 14–15), but encounters a problem retrieving the exact term. After a pause of 0.5 s, she provides a general formulation *xomer* “material” (line 16) and, after an additional 0.6 s, Kelsey suggests a potential candidate “plastic” (line 17), which Naomi confirms via nodding (line 18). After an additional hesitation (line 19), Naomi finally retrieves the professional term of the material of which such disposable utensils are made—polyurethane (line 20)—followed by “I think” to mark uncertainty (Ziv, 2016). Naomi then notes that she has learned about it at (textile) school (line 21), pointing at Kelsey again using her index finger (Image 10). By indicating where this knowledge was acquired, Naomi establishes her credibility and expertise on the subject. The utterance co-produced with the gesture does not include any reference to the second person, and the gesture appears to be employed to display epistemic authority. This epistemic status could be weakened by the speaker’s hesitations and expressions of uncertainty, making its reinforcement by the gesture particularly relevant in this moment of the interaction. Moreover, as in other occurrences, epistemic authority is further conveyed within a broad context of disaffiliation, where Naomi challenges Kelsey’s assertion that disposable utensils made of paper are preferable. Naomi then concludes that such material cannot decompose or be recycled (lines 22–24), coordinated with the Raised Index Finger gesture (Image 11), which is another means found to be associated with epistemic authority (Inbar, 2022).

In Excerpt 7, which is a continuation of the conversation between Kelsey and Naomi in which they discuss the use of disposable utensils, pointing at the addressee occurs five times. Naomi initiates a new telling regarding allegedly disposable utensils (lines 1–7). She first introduces this topic by stating that there are disposable utensils that are marketed as being degradable in compost (lines 5–7), ending with “continuing appeal intonation” (Du Bois, 2012, 5.3), which is also characterized as “try-marked” intonation (Sacks and Schegloff, 1979), an intonation contour which, in Hebrew, is designed to prompt a (minimal) response from the listener while signaling that there is more to be conveyed. Kelsey responds with *naxon* “right” (line 8), confirming that she is familiar with such utensils, upgrading her epistemic certainty by adding that she is also familiar with bags made from this material (line 9). Overlapping with Kelsey, Naomi projects surprise and unexpectedness by both uttering *ve* “and” (*cf.* Hopper, 2021) and employing a co-produced Raised Index Finger gesture (Image 14; Inbar, 2022), which she then transforms into a pointing gesture directed at Kelsey (Image 15) while strongly objecting to the information by stating *ze lo* “they are not” (line 11). Kelsey responds with her mouth wide open (Image 16), which is considered to be one of the components of a facial surprise display (e.g., Darwin, 1998; Reisenzein et al., 2012). Naomi then begins to explain how this substance can decompose (line 13), reproducing the index-pointing gesture directed at Kelsey to display epistemic authority. However, Naomi then hesitates and averts her gaze (line 14; Image 17), displaying a “thinking face” (e.g., Goodwin and Goodwin, 1986; Bavelas and Chovil, 2018). Interestingly, Naomi withdraws the index-finger pointing gesture as she starts to hesitate and reproduces it when she proceeds with her explanation (lines 15–16) (see discussion below on indicating the accomplishment of a cognitive process). Kelsey responds again with her mouth wide open (line 17). Naomi then tells Kelsey about her personal experience regarding such utensils, namely, that her mother put them into the regular compost, and they did not decompose (lines 20–21), deploying the index-finger pointing once again. By saying this, Naomi reinforces her epistemic authority by adding personal experience to her theoretical knowledge.

**Figure:**
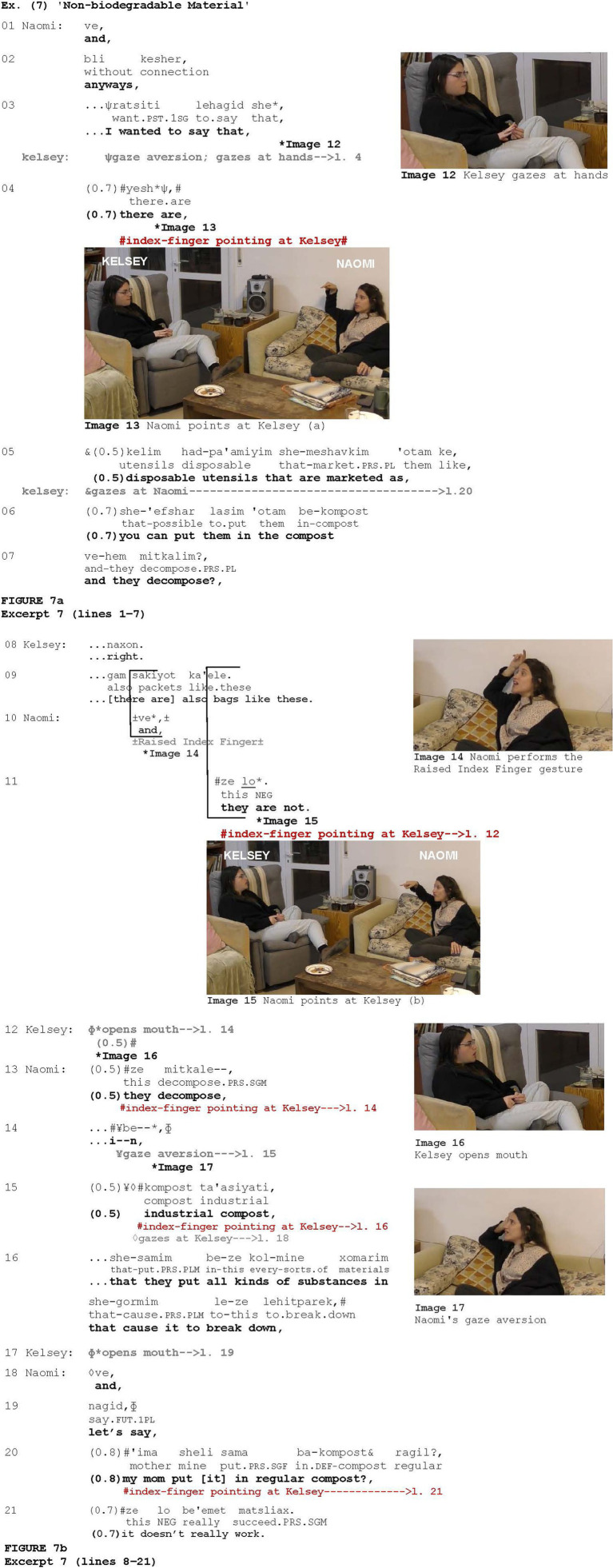


### 4.4 Gaining attention

In face-to-face interaction, attention can be signaled by a gaze directed at the speaker (e.g., Clark and Brennan, 1991). The findings suggest that after being disrupted, the interlocutor’s attention can be regained by the speaker pointing at their addressees. The current study revealed 15 instances of such addressee-directed pointing. The first occurrence of index-finger pointing in Excerpt 7 (Image 13) can be viewed as an example of the phenomenon. At the beginning of the excerpt, Naomi initiates a new telling regarding a particular kind of disposable utensil, prefacing her telling with a cluster of pragmatic markers (lines 1–3). During this prefacing, the recipient, Kelsey, averts her gaze (line 3) and starts to examine her fingers (Image 12). This type of behavior reveals a degree of reduction in her engagement in the interaction. It is plausible that, at this moment during the interaction, Naomi uses the pointing gesture as a device to attract Kelsey’s attention and to ensure her involvement. In other words, by pointing at Kelsey, Naomi could signal that she needs positive evidence of her attention. In fact, after Naomi points at her, Kelsey directs her gaze at Naomi again (line 5). Note that the context in which the gesture occurred cannot be characterized as opposition, nor is it one of the other specific contexts identified in this study in which index-finger pointing gestures occurred, except for those in which the interlocutor’s attention was diverted.

Excerpt 8, which is taken from a conversation between two friends, Lital and Eden, is another instance of using such pointing to attract the addressee’s attention to the upcoming talk. After a long pause (five seconds) during which there was no interaction between Eden and Lital—Eden was gazing at her cellphone and Lital was looking away—Lital initiates a turn saying *‘az* “so” (line 1), in continuing intonation, but Eden interrupts Lital by launching a course of action (*cf.* Sidnell, 2007) uttering *takshivi*! “listen!” (line 2), overlapping with Lital. The Hebrew verb *takshivi* “listen” can be considered as an attention-drawing device (e.g., Aijmer, 2010) and is used here in exclamatory intonation. Using *extreme case formulation* (Pomerantz, 1986), Eden then tells Lital that everyone is talking in the same way as she is (*kulam medabrim, kamoni!*), again using an exclamatory intonation (lines 3–4). Eden turns her gaze at Lital and Lital at Eden (line 4). Eden then produces the particle *hine* (line 5) which, in this case, indicates that Eden has visual access to the entity that serves as evidence for her previous statement (Shor et al., Forthcoming; Shor et al., Forthcoming), and points at Lital with her index finger (Image 18). Via this pointing, Eden obtains Lital’s attention toward her upcoming talk—reading her friend Gil’s WhatsApp message. Eden then points at her cellphone screen (Image 19), even though Lital cannot see the screen. While pointing at her cellphone screen, Eden starts reading the message in which Gil used the formulaic expression *‘avarti shinuy, ‘ani kvar* X “I’ve changed, I’m already X” (lines 8–11) that Eden had been using frequently, as she later explains (lines 13–15).

**Figure:**
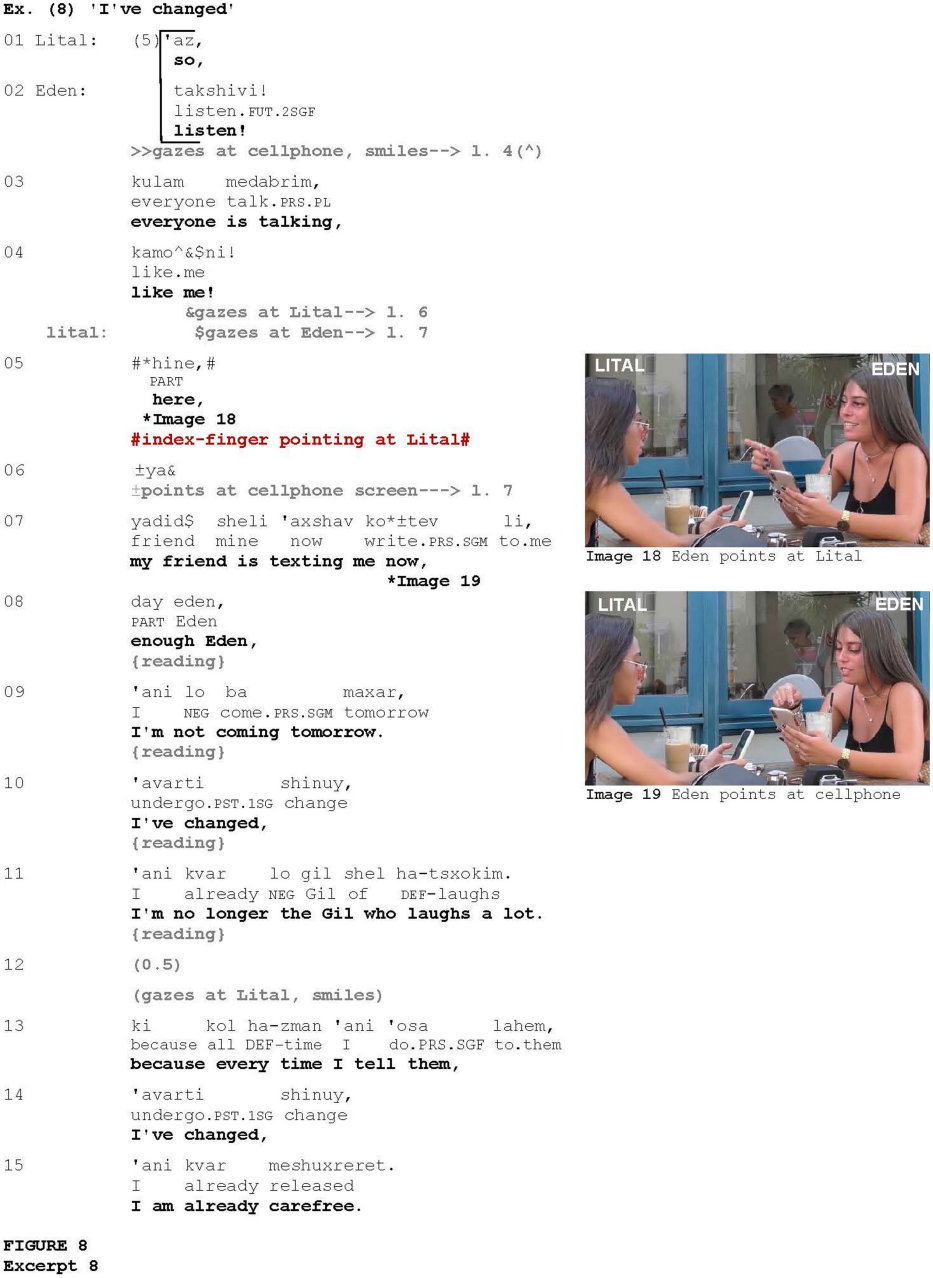


### 4.5 Indicating accomplishment of cognitive process

Index-finger pointing was also observed in contexts where the speaker had just undergone a certain cognitive process, often related to remembering. Conversational remembering has been claimed to be a systematic and joint activity that is performed for interactional purposes (e.g., Carranza, 2016; Hirst and Echterhoff, 2012; Middleton and Edwards, 1990). Carranza (2016) described three types of remembering sequences—*assisted*, *metacognitive*, and *spontaneous remembering*—all of which were attested in the current study as being associated with the index-finger pointing gesture directed at the addressee. One instance of index-finger pointing occurred in a *metacognitive remembering* sequence in which the speaker achieved remembering via the metacognitive strategy of a reflective question addressed to herself (“What else did she tell me?”). Another instance occurred in an *assisted remembering* sequence, in which a reminder was provided by the other participant. Yasui (2017) found similar cases in Japanese interaction, suggesting that the addressee-directed gestures may indicate that the source of the remembering was contained within what the current or prior speaker had just said, and were thus used as “touched-off” markers (Jefferson, 1987; Sacks, 1992). Finally, five remaining instances occurred in *spontaneous remembering* sequences, in which there were no visible reminders; that is, there was no talk that was designed to elicit remembering. Such cases are illustrated in the following two examples.

Excerpt 9 is taken from an interaction between two friends, Amit and Tom, who work as waitresses at the same place. The conversation revolves around a joint work shift. Amit begins a narrative about what happened on Thursday with their co-worker Noam, who was nervous and angry that day (lines 1–2, 5–7), and mentions that Tom was working on the same day (lines 3–4). After a stretch of talk (not shown) in which Amit recounts the chain of events during that shift, Tom suspends the discourse by noting that she does not remember Noam looking nervous (line 9). In overlap with the end of Tom’s utterance, Amit produces a click (line 10), projecting disaffiliation (Ben-Moshe and Maschler, Forthcoming). She then confirms that Noam was nervous, reinforcing her statement via the intensifier *mamash* “really” (line 11). Amit attempts to proceed with her talk (line 12), but Tom interrupts her again by upgrading her previous statement and saying that she actually does not remember anything about that shift (line 13). She then begins to recall what happened during that shift (line 14), but Amit produces another click (line 15), conveying impatience (Ben-Moshe and Maschler, Forthcoming), and again attempts to proceed with her talk (lines 16–17, 19). However, Tom interrupts Amit again, deploying the *change-of-state marker* (*cf.* Heritage, 1984, 2016) *ah* (line 18) to indicate that she has undergone a cognitive process, followed by spontaneously conveying remembering (line 20), overlapping Amit’s talk. The display of remembering is coordinated with pointing at Amit (Image 20). Amit reproduces her utterance for the fourth time, and finally manages to complete it (lines 21–22).

**Figure:**
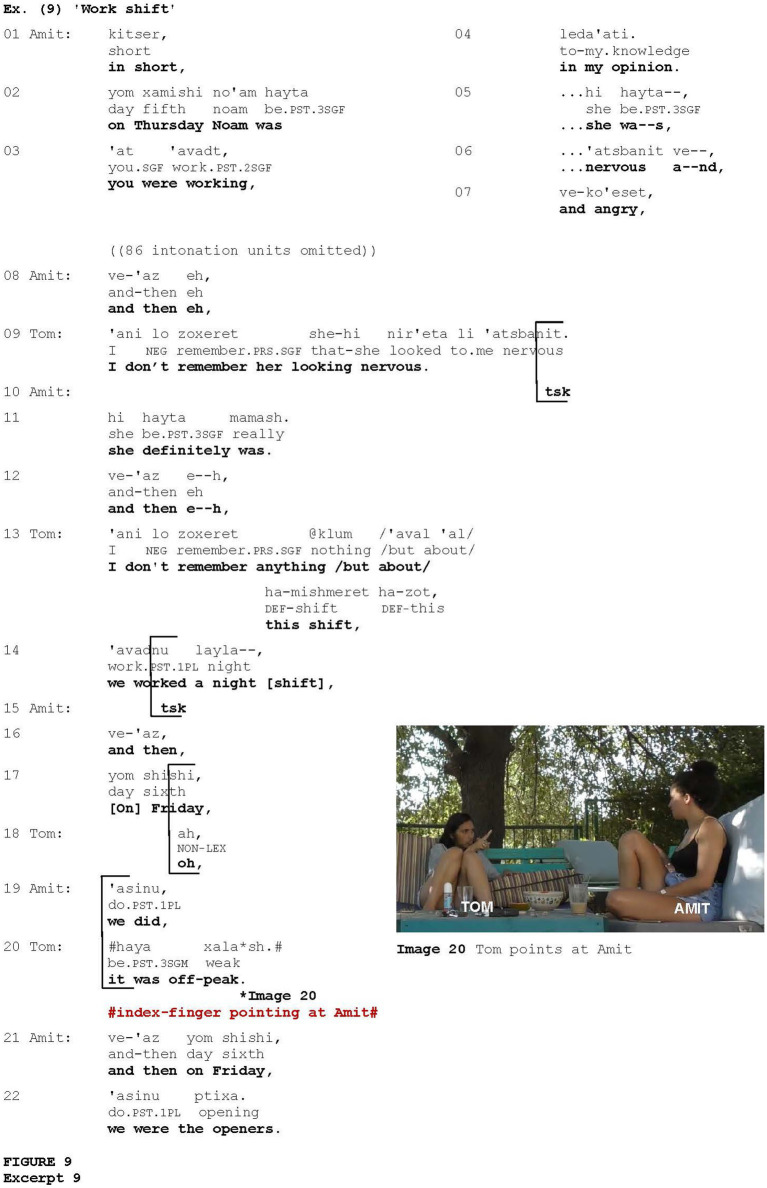


Another occurrence is illustrated in Excerpt 10, taken from an interaction in which Dotan attempts to explain how to get to a certain location in the city. After Dotan’s unsuccessful attempts to describe the exact location (not shown), Alex concludes that it is somewhere in the city center (lines 1–2). Dotan objects (lines 3–4) and, using a continuing appeal intonation, offers additional coordinates (lines 6–7), which Alex confirms via “Ok” (line 8) after a long pause. After another long pause (1.6), an inhalation, and the hesitation marker *e--hm* (lines 9–10), Dotan deploys the change-of-state marker *ah!* (line 11) to register either that he has undergone the cognitive process of remembering or that he has just figured out what is the best way to describe the direction. Dotan then produces the audible gesture of snapping his fingers (line 12), which, in various cultures, can be used to attract attention (e.g., Bowles, 2017; Christidou, 2018; Will, 2021). He then produces another hesitation marker *ne--hm* (line 13) coordinated with the index-finger pointing at Alex (Image 21), followed by offering another coordinate—the tunnel near Gan Sacher (lines 14–17).

**Figure:**
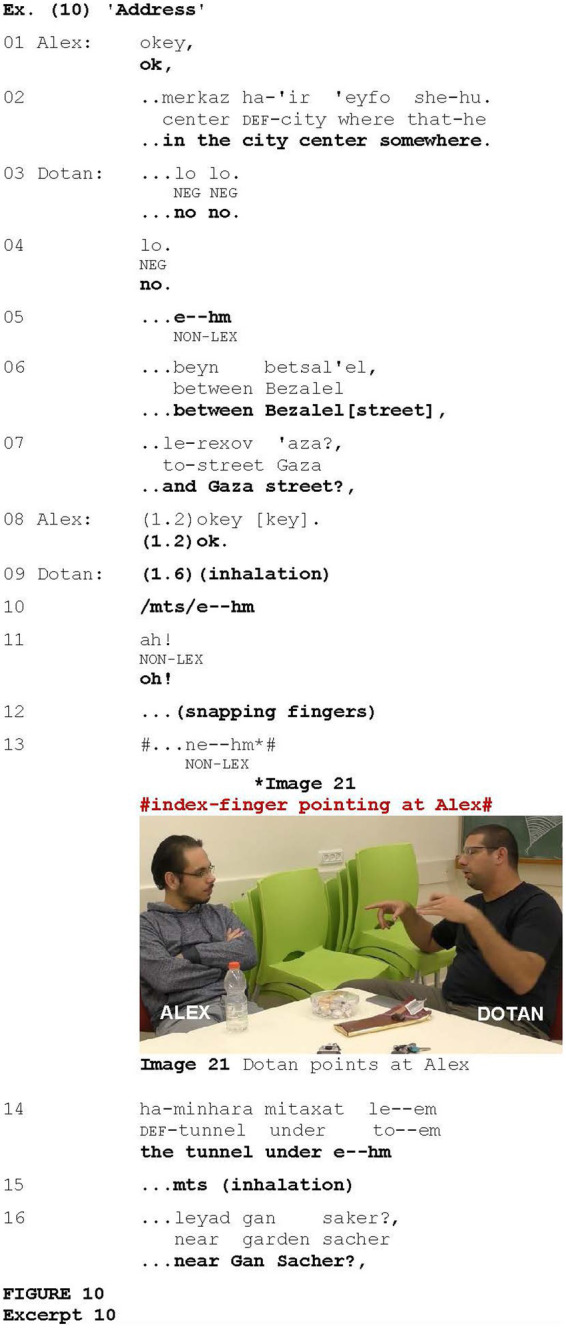


In such contexts, the gesture was often produced after pauses and hesitation markers on the part of the current speaker. Therefore, it is conceivable that the gesture was used to attract the interlocutor’s attention after the interlocutor could potentially have been distracted by such disfluencies, similar to the cases illustrated in Excerpts 7 and 8. In other cases, calling for attention to their remembering, speakers interrupted their co-participants, as in Excerpt 9. However, some occurrences of index-finger pointing at the addressee were associated with interruption and discourse suspension in other contexts, not necessarily those in which remembering was displayed (see Excerpt 11).

**Figure:**
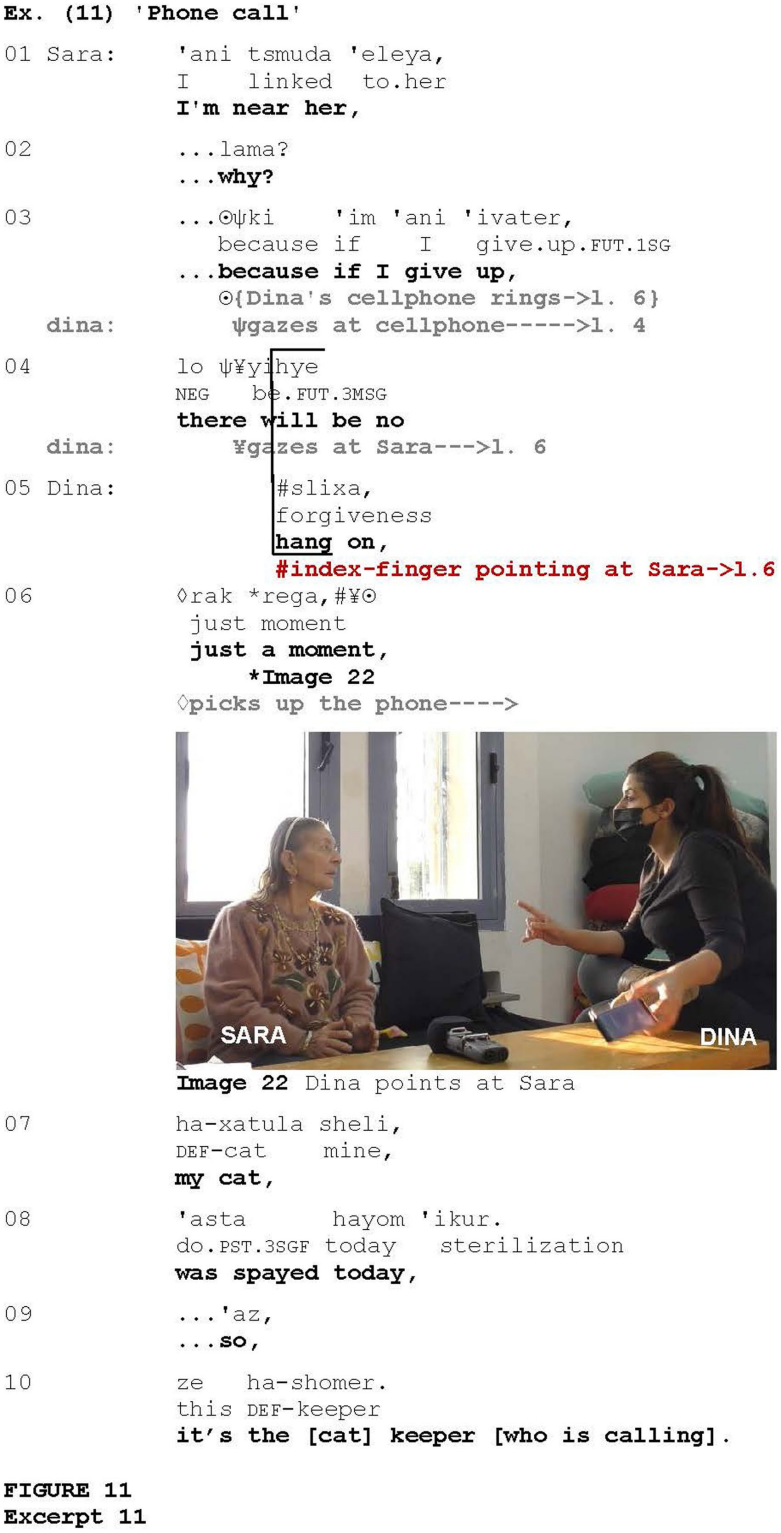


### 4.6 Interruption/discourse suspension

Another example of discourse suspension is illustrated in Excerpt 11, taken from a conversation between two acquaintances, Sara and Dina. Sara is a resident of the small settlement where Dina works. Sara begins to tell Dina about how she manages the treatment of her sick daughter. She says that she stays near her daughter constantly (line 1), and then starts to explain why (lines 2–3), but Dina’s cellphone suddenly rings.

As her cellphone rings, Dina interrupts Sara by deploying two discourse markers of *suspension* (e.g., Scott, 2002), *slixa* (*cf.* Inbar, 2022), and *rak rega* (*cf.* Bardenstein and Shor, 2019), which serve to indicate an attempt to stop others from speaking in order to gain or keep the floor. These discourse markers are coordinated with index-finger pointing at Sara (Image 22).

### 4.7 Eliciting a (minimal) response

The fact that pointing can be used when a speaker is attempting to elicit a response has been attested in conversation analytic studies (e.g., Bavelas et al., 1995), as well as in experimental studies (e.g., Holler, 2010). The data manifested numerous examples of ambiguous cases in which the gesture was employed in the context of eliciting a response and the utterance coordinated with the pointing included indexing the second person. However, the study revealed 13 occurrences of pointing at the addressee in the context of eliciting a response, in which the pointing was non-referential. Two such occurrences are illustrated in Excerpt 12. The excerpt is taken from a conversation between two friends, Orly and Sigal. Orly’s daughter is supposed to celebrate Bat Mitzvah—turning 12 years old, a landmark in Jewish tradition—and all the family is going to stay in a desert for this celebration.

**Figure:**
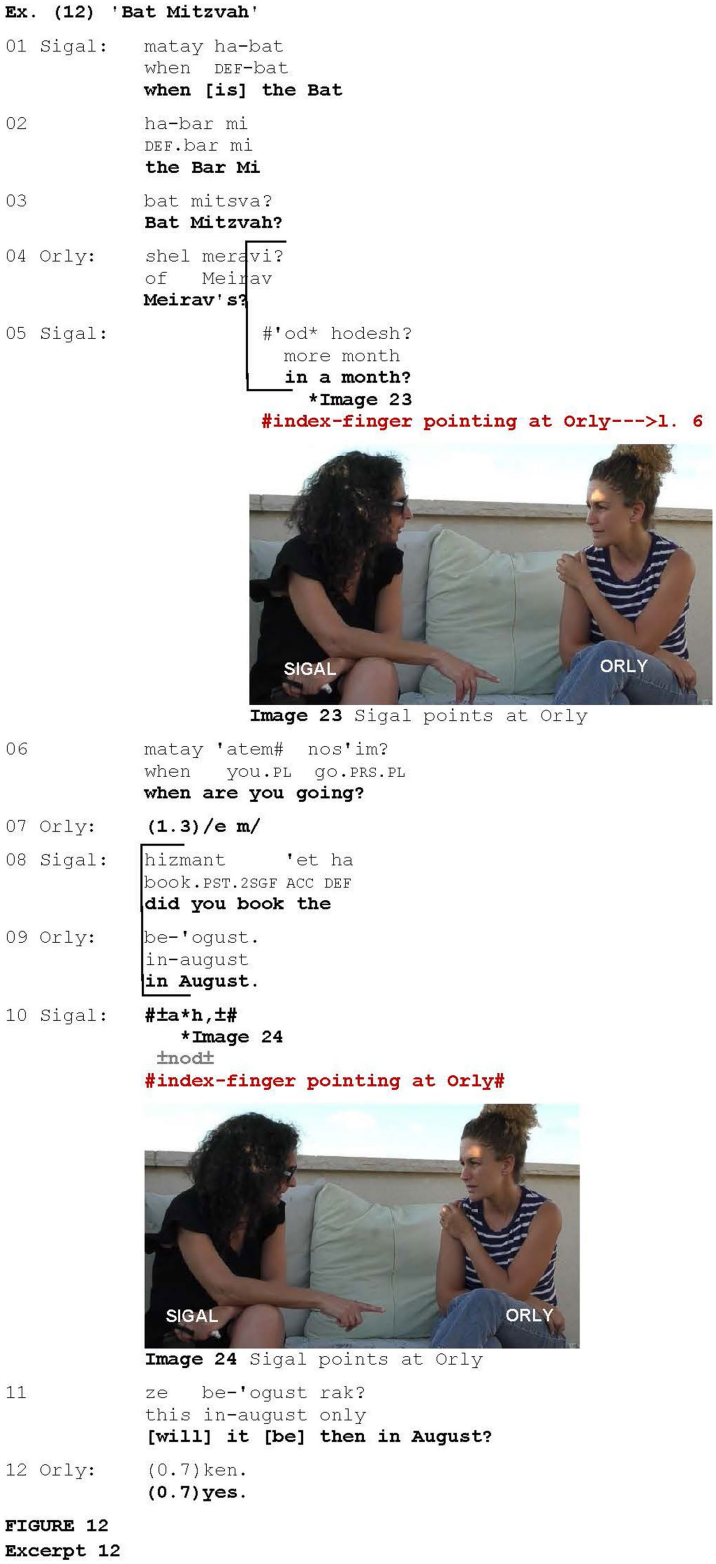


Coordinated with pointing at Orly with her index finger (Image 23), Sigal requests confirmation (*cf.* Ben-Moshe and Maschler, 2024) that the Bat Mitzvah of Orly’s daughter will take place in a month (line 5). Orly disconfirms saying that the celebration will take place in August (line 11). [Since the conversation was recorded in the beginning of June, it turns out that the celebration will take place later than was expected by Sigal.] Co-produced with another pointing at Orly (Image 24), Sigal deploys the change-of-state marker *ah* (line 12), followed by eliciting a minimal response from Orly deploying another confirmation request, this time framing the information requested for confirmation as unexpected via *rak* lit. “only” (line 13). Orly confirms via *ken* “yes” (line 14).

While in some examples of this variety, eliciting a response appeared to be combined with other contextual categories revealed in this study, in three cases, the gesture could also be interpreted as a device used to specify the addressee of an utterance in order to elicit their response (e.g., Bavelas et al., 1995; Enfield et al., 2007). These cases were observed only in interactions involving more than two participants, suggesting that the gesture is a versatile tool that adapts its functions to the dynamics of the interaction. Such an example is shown in Excerpt 13, which is taken from a conversation held during a family meal at Yair and Neta’s place with Yair’s parents. Prior to what is shown in Excerpt 13, the conversation revolved around salted fish which Yair’s parents do not like. Suddenly, Yair recalls a shared experience with his parents about their trip to Jordan where they were stuck without food, and someone brought them a canned fish.

Yair begins to recall a family trip to Jordan (lines 1–2). Then, Yair’s mother adds to the previous discussion concerning the salted fish, that smoked salmon is indeed tasty (line 3). Overlapping his mother, using an appeal intonation, Yair addresses his father with the request for confirmation that they had been traveling in the Dana Reserve (lines 4–5) during their trip to Jordan. This confirmation request is coordinated with the index-finger pointing at Yair’s father (Image 25) which is held until the information is confirmed via *ken* “yes” (line 6). In what follows, Yair and his father try to bring up memories of that experience whereas the family was stuck without food, and someone brought leftovers from some event (lines 7–15). In this example, the pointing occurs in the context of eliciting a response from Yair’s father, but since this interaction is multi-party, the gesture could also be interpreted as a device used to specify the addressee.

**Figure:**
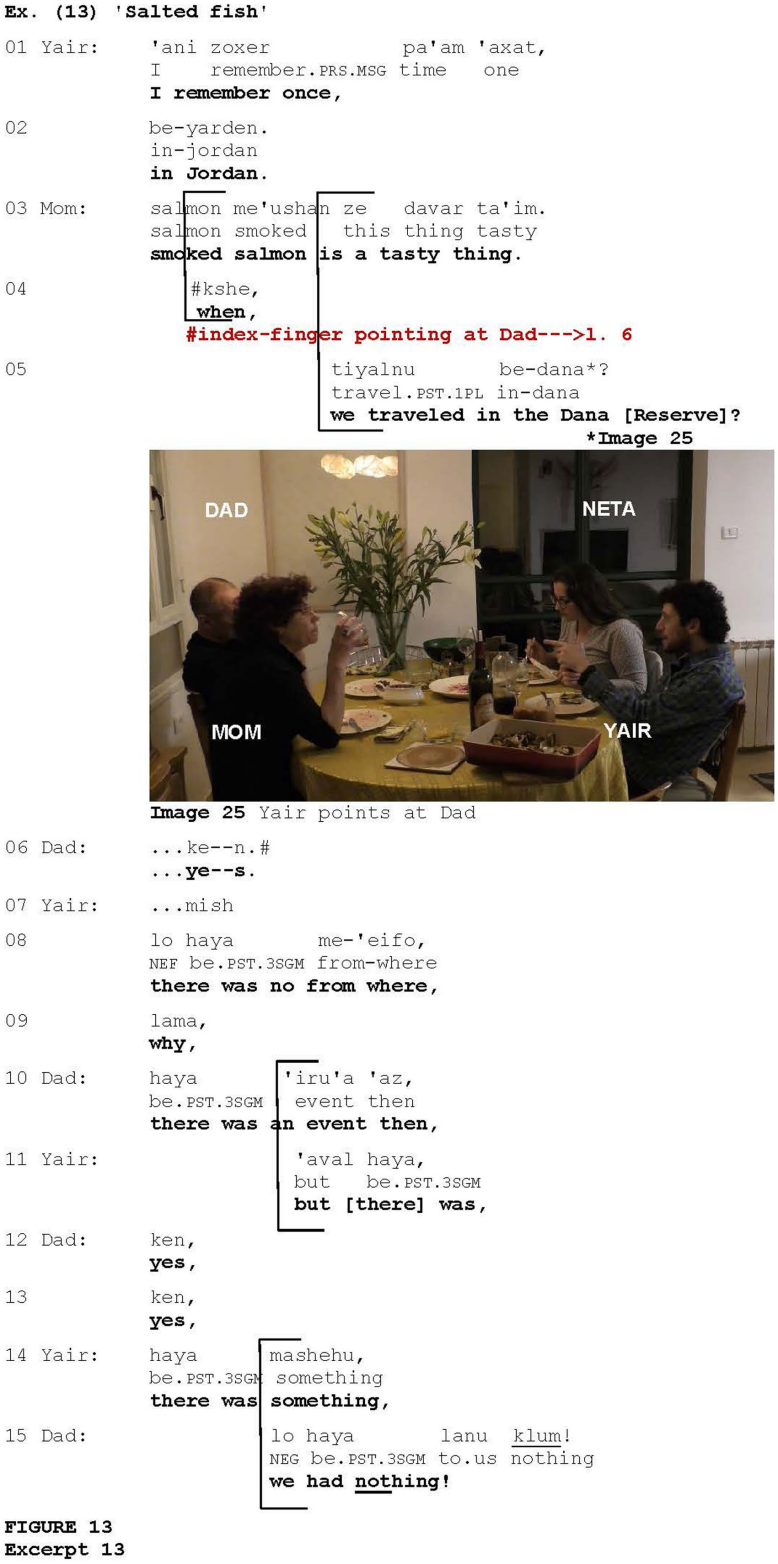


## 5 Discussion

The study showed that non-referential pointing directed at the addressee occurred in various contexts. In most of the cases (*N* = 41), the gesture occurred in contexts involving an action that did not support or endorse the co-participant’s stance or point of view. Some of them were disaffiliative contexts associated with dispreferred actions, such as disagreement, disconfirmation, and repair, while in others, the speakers conveyed information that was (assumed to be) contrary to the addresses’ expectations. In these contexts, the speakers typically produced and maintained a convergent status and stance of epistemic authority. Additionally, the gesture occurred when the addressee conveyed cues of lack of engagement in the interaction (*N* = 15), when they were indicating accomplishment of a cognitive process (*N* = 7), when the speakers attempted to elicit a (minimal) response from the addressee (*N* = 13), and when discourse suspension or interruption occurred (*N* = 5). The analysis of the examples shows that these contexts occasionally had an overlapping nature, making delineation among them challenging.

The pertinent question that emerges is why the gesture in question appears across these various contexts. It can be assumed that, similar to prototypical pointing, usually defined as a bodily movement toward a target in order to direct someone’s attention to it (e.g., Clark, 2003; Cooperrider, 2023; Cooperrider et al., 2018; Eco, 1976; Kendon, 2004; Kita, 2003), the non-referential index-finger pointing gesture directed at the addressee may primarily function as a mechanism for capturing attention. However, while the prototypical function that is commonly attributed to pointing gestures entails redirecting a listener’s attention to a referent (e.g., Cooperrider, 2023) that is most often presumed to be visual and present in the speech situation, the index-finger pointing at the addressee that was examined in this study mainly serves to attract the addressee’s attention to the pointer’s upcoming utterance. Interestingly, research in experimental psychology has shown that recipients are most likely to notice deictic gestures while interpreting speech (e.g., Langton and Bruce, 2000). The heightened awareness of these gestures suggests that they may play a crucial role in directing the recipient’s focus, thereby reinforcing the hypothesis that these gestures function as an attention-drawing device.

Existing research has revealed various motivations for capturing interlocutor’s attention, with this function being assigned to a range of devices (e.g., Aijmer, 2010; Atkinson, 1979; Brinton, 2001; Keenan and Schieffelin, 1976), whether verbal (e.g., address terms, locating directives or notice verbs such as *look* or *listen*, interrogatives, demonstratives, and imperatives) or non-verbal (e.g., pointing, touch, snapping gesture, (single) handclaps, the Raised Index Finger gesture, and throat clearing). One of the motivations for using attention-drawing devices may be a speaker’s sense that they are not being listened to (e.g., Aijmer, 2010; Romero Trillo, 1997). This motivation was particularly evident in contexts where the addressee conveyed cues of disengagement (Excerpts 7 and 8), in which the addressee-directed index-finger pointing gesture was employed effectively as an attention-drawing device. In these cases, the pointer sought positive evidence of the addressee’s attention in order to be reassured of their involvement in the interaction. Regaining attention was also relevant in the context of displaying the accomplishment of some cognitive process (Excerpts 9 and 10). In these cases, hesitations or disfluencies frequently arise, involving temporary suspension of flowing speech (e.g., Kosmala, 2021), thus potentially distracting the recipient’s attention.

The addressee-directed pointing gestures share additional features with other gestural and verbal attention-getters. The study showed that, similar to particular attention-getters, the gesture in question can be employed as an abrupt method of interruption (Excerpt 11) or as an attempt to elicit a (minimal) response from the addressee (Excerpts 12 and 13). The participants used the gesture to enter or intrude into the discourse in order to convey the content that they had just recalled or to request clarification or confirmation. In a similar vein, the Raised Index Finger gesture (Inbar, 2022; *cf.* Uskokovic and Talehgani-Nikazm, 2022) and various notice verbs (e.g., *look* and *listen*; Aijmer, 2010; Keenan et al., 1987), in addition to drawing attention to the message, were used to take the floor or to interrupt (e.g., Aijmer, 2010; Brinton, 2001).

Another reason for the use of attention-getting devices has been attributed to the need to emphasize an important part of the utterance in order to ensure that the recipient understands the message correctly (Romero Trillo, 1997; see also Brinton, 2001; Keenan et al., 1987). Bavelas et al. (1995) noted that speakers gesture toward another person to emphasize part of their speech, while Yasui (2023) observed that speakers may produce a pointing gesture to focus on one particular part of their utterance. Some scholars have pointed out that the need to draw attention to an utterance could be motivated by disagreements and that verbal attention-drawing devices could be used in argumentative contexts (e.g., Aijmer, 2010; Brinton, 2001). In fact, in the majority of the cases, the index-finger pointing at the addressee occurred in contexts that involved performing an action that did not align with or support the co-participant’s stance or perspective.

Moreover, this study has shown that disaffiliation was typically conveyed in tandem with establishing and sustaining the status and stance of epistemic authority. The association between epistemic authority and both verbal and non-verbal attention-drawing devices has been established in the literature. For example, Fairclough (2001) observed that speakers can assert their authority via the frequent use of the verbal attention-drawing device *look*. The connection between epistemic authority, opposition, and pointing has been highlighted in relation to the Raised Index Finger gesture (Inbar, 2022).

Another question to be addressed is what, nonetheless, is driving this direction of pointing. What motivates a speaker to point **at the addressee** in dyadic interactions, especially when the addressee’s reference is not indicated in speech? It can be assumed that this direction of pointing can be prompted by an **appeal** shared by all contexts, in that a favorable response or consideration is being sought from the addressee, rather than merely providing information. This appeal can encompass requests for engagement, clarification, suspension, or acknowledgment of the speaker’s perspective. The aim of engaging the addressee in the interaction brings non-referential pointing at the addressee closer to the category of summons (e.g., Pillet-Shore, 2018; Schegloff, 1968, 2002), designed to invite or prompt a response from a co-participant. However, the relation between the gesture and the category of summons warrants a more thorough examination.

## 6 Conclusion

This study focused on non-referential index-finger pointing at the addressee, which is a gesture that indexes the addressee for interactional purposes that extend beyond merely indicating a reference. The study revealed the contexts in which non-referential pointing at the addressee occurs in Hebrew talk-in-interaction. To elaborate on its pragmatic functions, the analysis was grounded in a detailed examination of the examples from each context, including the identification and analysis of the multimodal gestalts of which the gesture is part, as well as an exploration of the gesture’s formation and semiosis. The study proposed that the gesture may primarily serve as a means of capturing attention, showing that it shares several characteristics with other verbal and non-verbal attention-drawing devices. Revealing these characteristics and their interrelationships makes it possible to better understand the phenomena of non-referential pointing, pointing directed at the addressee, and attention-drawing devices in general.

The recurrent use of the gesture in particular contexts may plausibly lead to expectations from the addressee concerning other persistent aspects of the information provided, action accomplished, or stance taken by the pointer in such contexts. Consequently, these meanings may become conventionalized and recognizable via this gesture. For example, being frequently produced in contexts where a speaker opposes a co-participant’s stance—while also typically maintaining convergent status and a stance of epistemic authority—the gesture can be reanalyzed as a cue whereby conveying a negative or epistemic stance is recognized. By elaborating on such cases, this study contributes to the growing body of knowledge about multimodal stance-taking (e.g., Andries et al., 2023; Inbar, 2022; Inbar and Maschler, 2023; Newman et al., 2023; Shor and Marmorstein, 2022).

Furthermore, it has been indicated that both gestural and verbal deictic elements may evolve into stance markers via discourse deixis (e.g., Inbar, 2022; Shor and Inbar, 2019). For example, Heine and Kuteva (2002) note that, in various languages, demonstratives provide fertile ground for grammaticalization processes that can lead to the creation of various grammatical elements, including focus particles. These studies reinforce the conceptual link among deixis, focus, and stance. This connection is also highlighted herein.

The study showed that the addressee-directed pointing gesture may entail the blending of two or more semantic or functional categories to form a multifaceted hybrid sign. Moreover, the gesture can be accounted for from different perspectives, while various factors (e.g., morphological differences, seating arrangements, the number of participants) may have an impact on its employment. To move toward a more generalized analysis of the phenomenon and to develop distributional rules, the analysis should be expanded by considering new data. Finally, further research is needed to explore potential cultural differences, variations in genre and gender, and a more detailed examination of the similarities and differences among other verbal and non-verbal means used to attract attention in interaction.

## Data Availability

The data analyzed in this study is subject to the following licenses/restrictions: The datasets presented in this article cannot be publicly available for privacy reasons. Requests to access these datasets should be directed to Anna Inbar, inbara9@gmail.com.

## Footnotes

^1^For examples of such ambiguous cases see Excerpts 1 and 6, line 8.

^2^In such cases, the assignment to a category was based on the salient features that were reflected in other gestural or verbal means. Thus, the distribution presented above is provided to offer a general impression of the data and should not be interpreted as a precise representation.

## Appendix

Transcription conventions

(Following Chafe, 1994; Du Bois et al., 1992; Du Bois, 2012 and adapted for Hebrew (Maschler, 2017))

.. – perceptible pause of less than 0.1 s

... – average pause (0.1 ≤ x < 1.0 s)

.... – pause (1.0 ≤ x < 1.5 s)

.... – pause (1.5 ≤ x < 2.0 s).

(3.56) – measured pause of 3.56 s.

, − comma at end of line –mid-level, mid-rise, mid-fall intonation, regularly understood in Hebrew as ‘more to come’

. – period at end of line – low fall intonation, regularly understood in Hebrew as final.

? – question mark at end of line – high rising intonation, regularly understood in Hebrew as.

final and seeking response from interlocutor.

?, − question mark followed by comma – rising intonation, regularly understood in Hebrew as projecting ‘more to come’ while seeking response from interlocutor.

! – exclamation mark at end of line – final exclamatory intonation.

ø – lack of punctuation at end of line – a fragmentary intonation unit, one which never reached completion.

-- two hyphens – elongation of preceding sound.

underlined syllable – primary stress of intonation unit.

**bold**faced syllable – secondary stress of intonation unit.

@ – a burst of laughter (each additional @ symbol denotes an additional burst).

square bracket to the left of two consecutive lines indicates.

beginning of overlapping speech, two speakers talking at once.

inverted bracket + alignment such that the right of the top line.

is placed over the left of the.

bottom line indicates latching, no interturn pause.

Musical notation as necessary: e.g., *acc* – *accelerando* (progressively faster).

(in regular brackets) – nonverbal action constituting a turn.

{in curly brackets} – transcriber’s comments.

‘– uninverted quotation mark indicates the glottal stop phoneme.

- one hyphen – bound-morpheme boundary.

/words within slashes/ indicate uncertain transcription.

Transcription of embodied conduct.

(following Mondada, 201**9**)

# # Descriptions of embodied conduct are delimited in between symbols.

± ± Two identical symbols (one symbol per participant and per type of conduct) are synchronized with corresponding stretches of talk.

#----> Described embodied conduct continues across subsequent lines

---- > # until the same symbol is reached.

........ Action’s preparation.

--------- Action’s apex is reached and maintained.

#----- > l.12 Described embodied conduct continues until line 12 of transcript.

#----->> Described embodied conduct continues beyond end of excerpt.

> > −---- Described embodied conduct begins before the excerpt’s beginning.

* Exact position in the utterance in which a video caption was made.
